# Late infection of a breast prosthesis with *staphylococcus aureus* in a healthy woman: a case report

**DOI:** 10.1093/jscr/rjac067

**Published:** 2022-03-07

**Authors:** Rami Dartaha, Muhamad Zakaria Brimo Alsaman, Afnan W M Jobran

**Affiliations:** 1 Rambam Hospital, Haifa Private Clinics, Haifa, Palestine; 2 Faculty of Medicine, University of Aleppo, Aleppo, Syria; 3 Faculty of Medicine, Al Quds University, Jerusalem, Palestine

**Keywords:** breast, prosthesis, infection, *Staphylococcus aureus*, case report

## Abstract

Infection following breast augmentation is a rare event with an incidence rate of 1–2.5%. Late onset infections following breast augmentation are very rare. Herein, we present a case of breast implant infection in a 29-year-old female patient who underwent a bilateral augmentation mammoplasty with a silicone gel prosthesis. After 8 uneventful post-operative years, she presented with right-sided signs of breast infection. She initially treated medically but without improvement. Then, she underwent surgical washout and debridement with removal of the bilateral breast implants. Culture demonstrated *Staphylococcus aureus*. The clinical history and management of this unusual case are described. Because most of the infections occurs in the first few weeks after augmentation mammoplasty, there is a paucity of data about late onset infections. The most common cultured organism in the early infection is *S. aureus*.

## INTRODUCTION

Augmentation mammoplasty is a common surgical procedure with breast implants. It is used for reconstruction after mastectomy, breast enlargement and correction of asymmetries [1]. The incidence rate of breast implant infection ranges from 1 to 2.5% [1], but the incidence that follows breast reconstruction has been reported as much higher and this may be attributed to factors such as tissue scarring and skin atrophy from radiation [2]. Because most of breast implant infection occurs in the first month after implantation, there is little data about late onset infections [2]. Therefore, it has bimodal fashion, during the acute post-operative period (6 days to 6 weeks after surgery) or late onset infection (more than 6 months after surgery). Early onset infection of breast implant is typically associated with fever, breast pain, erythema and purulent fluid or drainage. However, some patients may have systemic signs and symptoms of infection such as toxic shock syndrome which is usually caused by *Staphylococcus aureus* that is acquired during surgery. Late onset infections occur several months to years after implant and are rare and usually resulting from secondary bacteremia due to infection at another site [3, 4]. The most common isolated organisms from breast implant infection are *S. aureus* and coagulase-negative staphylococci. *Staphylococcus epidermidis* is the most frequent coagulase-negative Staphylococcus species. *Pseudomonas aeruginosa* is the second most commonly identified pathogen. Other pathogens that have been involved in breast implant infection include klebsiella, streptococci A and B, *Proteus mirabilis*, enterobacteria and mycobacteria [5, 6, 7, 2]. Breast contains endogenous flora that normally habitat in the mammary duct, which is mostly *S. epidermidis*, and these bacteria may gain access to deeper tissue during breast manipulation during operation, but these mostly responsible for acute breast implant infection [8]. Here, we present a case of breast implant infection in an otherwise healthy patient, 8 years after augmentation mammoplasty. *Staphylococcus aureus* was cultured from the clinical specimen. Although there were no identifiable predisposing factors for the introduction of the organism.

## CASE PRESENTATION

**Figure 1:**
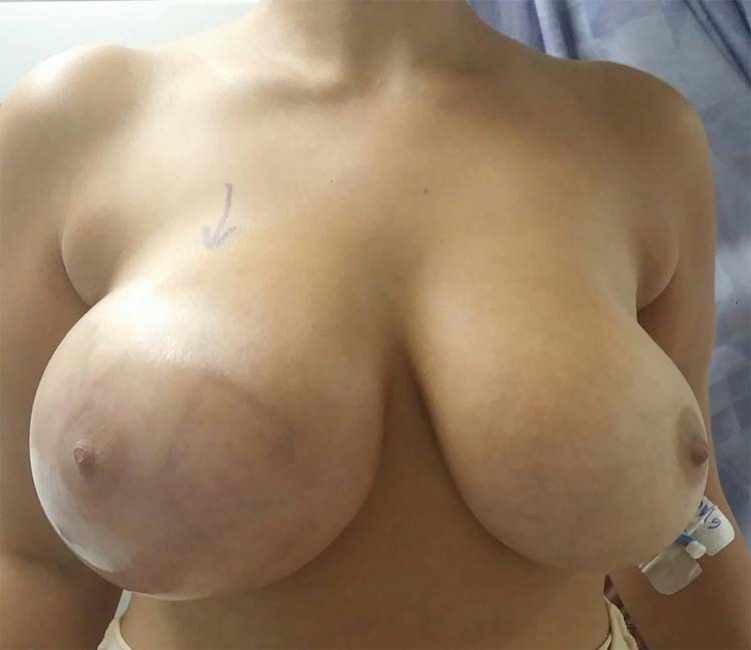
A 29-year-old woman is shown 8 years after right breast aesthetic mammoplasty with silicone gel implants. There is right breast swelling and erythema.

A 29-year-old white woman underwent a bilateral augmentation mammoplasty with silicone gel prosthesis 8 years ago. The patient presented with 2 weeks of acute onset right-sided breast swelling, redness and pain (Fig. 1). She denied history of breast discharge, trauma and previous history of the same complaint. She was given (Augmentin 875-mg tablets, BID) with no improvement. She was satisfied with size of her breasts and was generally pleased with implants. On physical exam, the patient was febrile 38,8. The right breast was swollen, erythematous, warm and tender. There was no discharge or sinus tract noted, and there were no other palpable masses. The infra-mammary incision was well healed and was not specifically involved in the inflammatory process. The left breast was normal in shape and without tenderness or hotness. WBC count was 23,9 with lift shift. Urinalysis and chest X-ray were normal.

**Figure 2:**
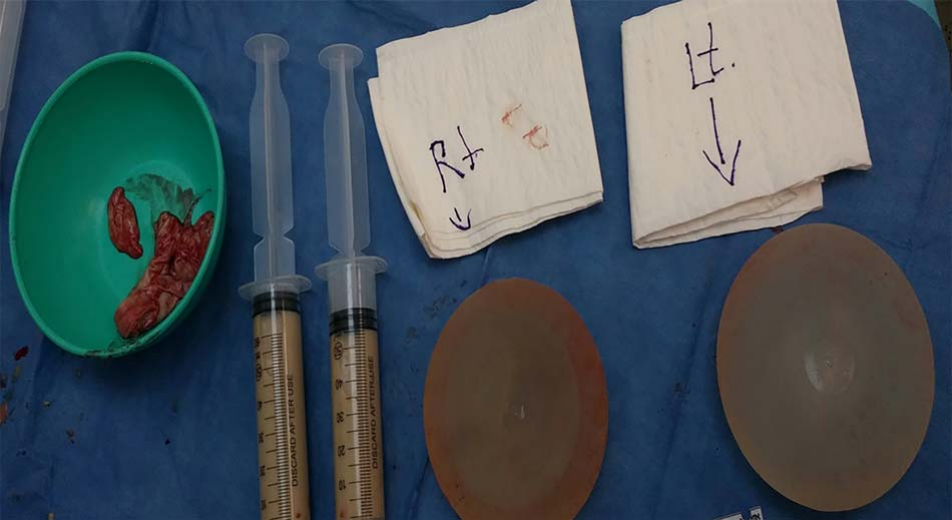
View of both breast implants after capsulectomy, specimen was taken from right breast implant and tissue for biopsy and specimen for culture.

Breast ultrasound showed small amounts of fluids around the implant without pus discharge. Therefore, patient was admitted to our department for IV antibiotics, and she was given Augmentin and ciprofloxacin with minimal improvement. The patient continued to have constant pain with signs of local breast inflammation given that the patient underwent debridement operation.

**Figure 3:**
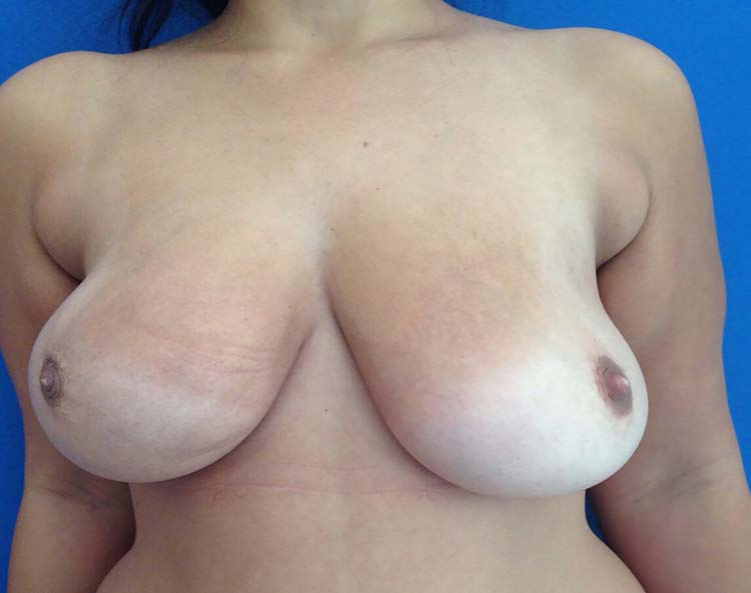
View of both breasts 3 months after surgery without signs of infection.

Intra-operatively, there was aggressive inflammatory process in the right breast due to implant, sub-glandular tissue in the right side was surrounded by purulent material (Fig. 2). The implant was intact. Intra-operative aerobic and anaerobic cultures were performed. Biopsy was performed from the capsule and breast tissue; copious irrigation of the cavity was performed, followed by hemostasis. Closed suction drains were left in place, and the cavity was closed loosely with absorbable suture in the deeper tissue and nylon skin sutures. The patient was maintained on intravenous antibiotics (Augmentin and ciprofloxacin). The patient was discharged home on oral Augmentin and ciprofloxacin. The drains were removed when the drainage was minimal. Cultures from the right breast revealed staph aureus, which was sensitive to antibiotics treatment. Three months after surgery, right breast incision was well healed without evidence of infection and the patient has remained pleased with her result (Fig. 3).

## DISCUSSION AND CONCLUSIONS

Breast augmentation with implants is one of the most popular aesthetic surgical procedures. The most commonly used implants worldwide is silicone gel-filled implant [9, 10].

*Staphylococcus aureus* is an important pathogen responsible for a broad range of infections ranging from benign skin infections to life threatening conditions such as osteomyelitis and endocarditis. It is also a commensal pathogen colonizing 30% of human population. *Staphylococcus aureus* usually is a cause of early onset breast implant infection, less likely to be the causative agent in late onset infection [6].

This case deals with a previously healthy woman with no underlying disease or any with no recent history of taking immunosuppressant medications, no evidence of remote infection and no obvious opportunities for introduction of organisms [2]. The origin of infection in breast implant is difficult to determine, but there are some possible sources that include the patient’s skin or mammary duct, contaminated saline, contaminated implant, the surgery itself and sometimes hematogenous spread [4]. Another cause of late onset infection attributed to low virulence pathogens such as Mycobacterium species that were present at time of operation, but it take time to develop signs and symptoms, but less likely with *S. aureus* which is high virulence organism as in our case [11].

Most studies related to breast implant infection on the peri-operative factors that may responsible for causation of infection such as use antibiotic prophylaxis, surgical approach with manipulation during surgery and presence of hematoma. The exceptionally long period after breast implant eliminates the role of these factors. A study suggested that hematogenous spread of bacterial infections to breast implants from distant sites may play a crucial role for developing late onset breast implant infections [12].

Patients who undergoing reconstructive breast surgery are more likely to accept complications such as breast implant infection, in addition to antibiotic therapy, additional surgeries and other therapy in comparison to patients who undergoing aesthetic breast surgery [9].

One large study suggested that silicone gel implant might be associated with later onset implant infection as in our case than saline implant [13].

Late onset implant infection usually has severe presentation as compared with early onset infection. Severe presentation includes cellulitis, purulent discharge, implant exposure and sometimes systemic signs and symptoms. Patients with mild breast implant infection usually respond to conservative treatment with implant salvage, which include mechanical irrigation, topical and systemic antibiotics, capsulectomy and immediate replacement of the implant at the time of surgical intervention. In opposite patients with severe breast implant infection, they do not respond to conservative treatment and need implant exploration as in our case [9, 2].

Patients with breast implant who are exposed to potential bacterial inoculation and bacteremia should be treated with systemic antibiotics to prevent late breast implant infection and subsequent complications [12].

Infection in aesthetic breast augmentation occurs on rare occasions with an overall incidence about 1–2.5%. Because most of the infections occurs in the first few weeks after augmentation mammoplasty, there is a paucity of data about late onset infections. The most common cultured organism in the early infection is *S. aureus*.

We described a case of an infected breast prosthesis 8 years after augmentation mammoplasty, in which purulent material and aggressive inflammatory process in the right breast were found. As in many reported cases, no definite cause was found.

Hematogenous spread of bacterial infections to breast implants from distant sites may play a role for developing late onset breast implant infections. Therefore, we suggest early treatment for any bacterial infection in the breast implant prosthesis patients.

## AUTHORS’ CONTRIBUTIONS

R.D. and A.W.M.J.: analyzed and interpreted the patient data, wrote the manuscript, revision.

M.Z.B.A.: revision critically and the corresponding author.
